# Afferent Neurons of the Zebrafish Lateral Line Are Strict Selectors of Hair-Cell Orientation

**DOI:** 10.1371/journal.pone.0004477

**Published:** 2009-02-18

**Authors:** Adèle Faucherre, Jesús Pujol-Martí, Koichi Kawakami, Hernán López-Schier

**Affiliations:** 1 Laboratory of Sensory Cell Biology & Organogenesis, Centre de Regulació Genòmica, Doctor Aiguader, Barcelona, Spain; 2 Division of Molecular and Developmental Biology, National Institute of Genetics, and Department of Genetics, The Graduate University for Advanced Studies (SOKENDAI), Mishima, Shizuoka, Japan; Katholieke Universiteit Leuven, Belgium

## Abstract

Hair cells in the inner ear display a characteristic polarization of their apical stereocilia across the plane of the sensory epithelium. This planar orientation allows coherent transduction of mechanical stimuli because the axis of morphological polarity of the stereocilia corresponds to the direction of excitability of the hair cells. Neuromasts of the lateral line in fishes and amphibians form two intermingled populations of hair cells oriented at 180° relative to each other, however, creating a stimulus-polarity ambiguity. Therefore, it is unknown how these animals resolve the vectorial component of a mechanical stimulus. Using genetic mosaics and live imaging in transgenic zebrafish to visualize hair cells and neurons at single-cell resolution, we show that lateral-line afferents can recognize the planar polarization of hair cells. Each neuron forms synapses with hair cells of identical orientation to divide the neuromast into functional planar-polarity compartments. We also show that afferent neurons are strict selectors of polarity that can re-establish synapses with identically oriented targets during hair-cell regeneration. Our results provide the anatomical bases for the physiological models of signal-polarity resolution by the lateral line.

## Introduction

The lateral-line system of fishes and amphibians serves to detect hydrodynamic variations and water currents [1]–[3]. The organs of the lateral line respond to low-frequency mechanical signals and have been experimentally implicated in a number of behaviors, such as schooling, rheotaxia, prey capture, and obstacle and predator avoidance [4]–[6]. The lateral line plays an important role in motor reactions, and the animal's dependence on this sensory system is greatest when vision is limited [7]–[8]. The functional units of the superficial lateral-line system are called neuromasts, which occur freestanding on the surface of the animal. In the zebrafish larva, each neuromast contains 20 to 30 mechanosensory hair cells and a similar number of non-sensory supporting cells [9]–[12]. Approximately 20 bipolar afferent neurons receive synaptic input from the hair cells of each major branch of the lateral line. These neurons coalesce in small cephalic ganglia, projecting fibers into a rostrocaudal column in the hindbrain [9], [13]–[14]. Efferent neurons that innervate hair cells of the lateral line locate their cell bodies in three distinct nuclei in the central nervous system, distant from the afferent ganglia [9], [15].

Sensory hair cells show a characteristic planar polarization evidenced by the eccentric localization of a kinocilium and its surrounding chevron-shaped stereocilia. A mechanical deflection of the stereocilia towards the kinocilium opens transduction channels to depolarize the cell's plasma membrane, whereas a deflection away from the kinocilium hyperpolarizes the cell [16]. Therefore, the axis of morphological polarity of the stereocilia corresponds to the direction of excitability of the hair cells, which in turn enable animals to determine the vectorial component of a sound [17]–[19]. In the mammalian cochlea all hair cells are oriented identically across the plane of the tissue. Thus, the discrimination of the direction of mechanical stimulation is performed by the sensory epithelium. However, each neuromast of the lateral line consists of two intermingled populations of hair cells, equal in number, whose stereocilia are oriented at 180° relative to each other [11]–[12], [17], [20]. This organization creates ambiguity in the direction of the mechanical stimulation. How is the stimulus-polarity problem solved in this system? Pioneering electrophysiological analyses in *Xenopus* and in cichlid fish demonstrated that lateralis afferents respond differentially to the direction of hair-cell stimulation, supporting the hypothesis that these neurons can discriminate the polarity of the stimulus [21]–[22]. However, alternative views suggest that afferent fibers show no directional sensitivity [23]–[25]. Therefore, how some animals compute bidirectional mechanical stimulation remains controversial. In the present study we use transgenic zebrafish, molecular markers, live imaging and genetic mosaics to address this question.

## Results

### The HGn39D enhancer-trap marks the afferent neurons of the lateral line

**Figure 1:**
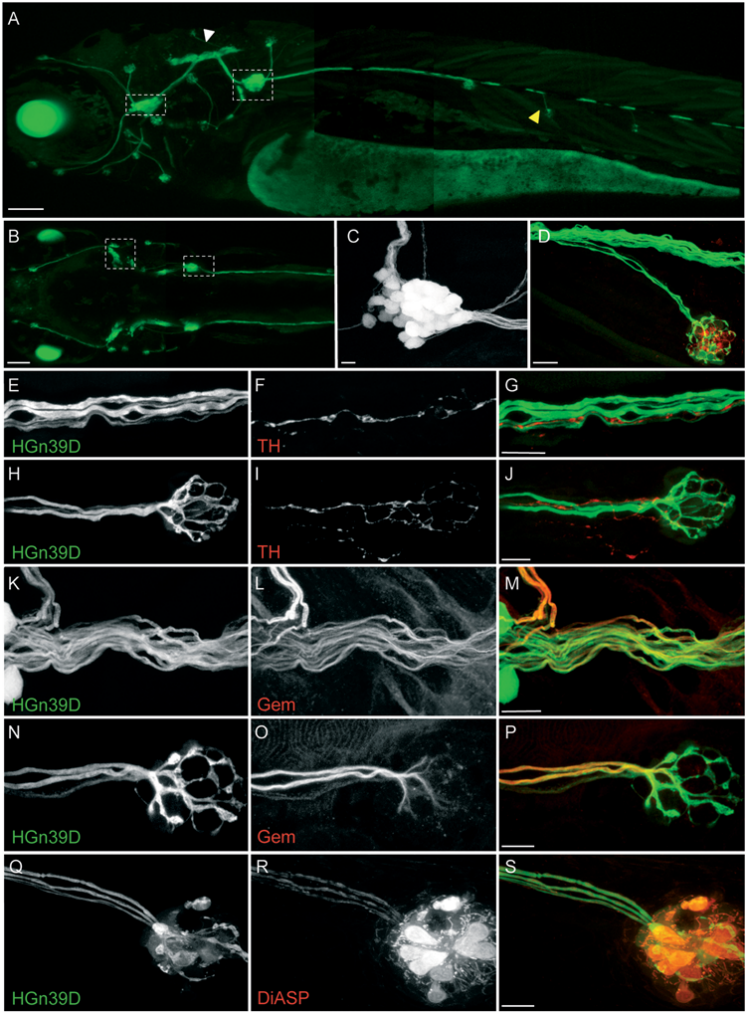
HGn39D transgenic zebrafish express GFP in the lateral line afferent neurons. (A–C) GFP expression pattern in a 4 dpf HGn39D transgenic fish. Maximal projections of lateral- (A) and top-views (B) are depicted. White and yellow arrowheads point to the neuronal projections to the hindbrain and to the neuromast respectively. Dashed frames indicate the lateral line ganglia in A and B. (C) Maximal projections of the posterior lateral line ganglion; approximately 25 somata can be counted. (D) Maximal projection of an HGn39D innervated neuromast immunolabeled with anti-HCS1 antibody (red). (E–P) Anti- tyrosine hydroxylase (TH) and Gem25.2/Cav1.3a (Gem) immunostainings were performed on 7 dpf HGn39D fish. (E,H,K,N) HGn39D GFP expression; (F,I) anti-TH antibody labels the efferent neurons; (L,O) Anti-Gem antibody labels the afferent neurons. Maximal projections of the posterior lateral line nerve (E–G, K–M) and of the terminal neuromast of the posterior lateral line (H–J, N–P) are depicted. (G,J,M,P). Overlays of HGn39D GFP expression and specific immunostainings. (Q–S) DiASP is trans-synaptically transported into the HGn39D afferent neurons. (Q) HGn39D GFP expression; (R) DiASP incorporation; (S) Overlay of HGn39D GFP expression and DiASP incorporation. Scale bars: 100 µm (A, B) and 10 µm (C–S).

In the zebrafish, the lateral-line system develops early in embryogenesis from bilateral cephalic neurogenic placodes, which generate precursors of the afferent neurons and a motile primordium precursor of neuromasts. Posterior lateral-line primordial cells migrate collectively along the horizontal myoseptum, guiding the extension of axons of the afferent neurons whose cell bodies coalesce into a postotic ganglion [9]–[10], [26]–[27]. Lateralis afferent neurons have been often revealed using molecular markers on fixed material, or by random filling of fibers with neuronal tracers [9], [14], [26]. These methods are not amenable to long-term imaging of the full architecture of lateralis afferents in living specimens. We performed a large-scale enhancer-trap screen and generated a transgenic line named HGn39D that expressed the GFP in small groups of neuron-like cells that extended fibers around the head and along the trunk of the zebrafish embryo and larva (Figure 1 A) [28]. The GFP is also strongly expressed in the lens of the eye. Using laser scanning confocal microscopy, we observed that GFP-expressing cells form ganglia at the anterior and posterior sides of the ear (Figure 1 A–C), away from the three efferent nuclei in the di- and rhomb-encephalon [29]. GFP-expressing cells project axons to the central nervous system that form a rostrocaudal column at the level of the hindbrain and fibers along the entire trunk of the animal (Figure 1 A). In the periphery, GFP-positive fibers terminate in elaborated dendritic arbors whose thin extensions contact hair cells in all neuromasts (Figure 1 D). Therefore, the shape and organization of these GFP-expressing cells are indicative of them representing afferent neurons of the entire lateral-line systems. We probed their identity at the molecular level by performing immunohistochemical staining on HGn39D transgenics with an antibody against tyrosine hydroxylase, a marker of lateral-line efferent neurons [30]. This antibody marked the efferents in the main nerve fascicle and at the level of the neuromasts (Figure 1 E–J), but did not stain GFP-positive cells suggesting that they are not efferent neurons (Figure 1 G and J). To confirm their identity, we also stained samples with the antibody Gem25.2, which has been shown to label lateralis afferents in the zebrafish. In all cases, the signal from Gem25.2 co-localized with that of the GFP (Figure 1 K–P) [31]. Finally, we employed the cationic fluorophore 4-(4-(diethylamino)styryl)-*N*-methylpyridinium iodide (4-Di-2-ASP or DiASP) which has a broad emission spectrum and fluoresces orange. When exposed to zebrafish larvæ, DiASP is taken up by hair cells of the lateral line and later transported trans-synaptically to afferent neurons [11], [32]. After incubating HGn39D larvæ in DiASP, all GFP-positive neurons were orange, and no other neuron-like cell incorporated the dye (Figure 1 Q–S). From these results, we conclude that GFP in the HGn39D enhancer-trap line specifically reveals the whole population of lateralis afferent neurons.

### Two or more afferent neurons contact hair cells in each neuromast of the lateral line

**Figure 2:**
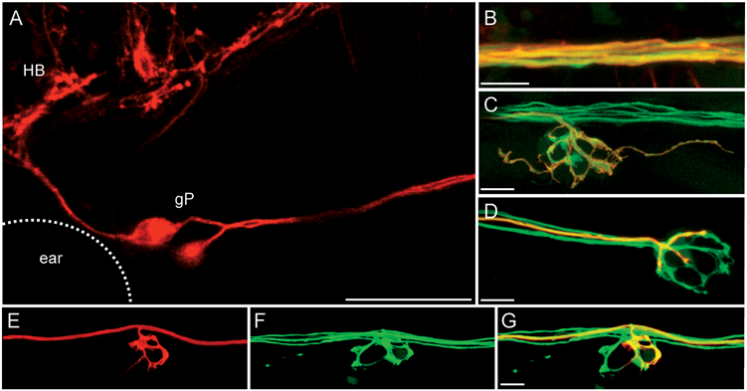
The HuC promoter drives the expression of mem-TdTomato in the afferent neurons. Maximal projections of HGn39D fish injected with HuC∶mem-TdTomato are depicted. (A) HuC promoter drives the expression of mem-TdTomato in the soma of the afferent neurons (HB: hinbrain, gP: Posterior lateral-line ganglion). (B–C) mem-TdTomato is expressed in GFP-positive neurons of HGn39D fish at the level of the defasciculated fibers (B) and of the dendrites beneath the neuromast (C). (D) mem-TdTomato is expressed in a single neurons. (E–G) Injection of HuC∶mem-TdTomato allows the mosaic expression of red neurons. A single red neuron (E) can be observed in HGn39D (F). (G) Overlay of HGn39D GFP and mem-TdTomato. Scale bars: 50 µm (A) and 10 µm (B–G).

We observed that two or more afferent fibers de-fasciculate from the main nerve towards each neuromast in HGn39D (Figure 1 D, N, Q). The tight fasciculation of the axons does not allow their individual resolution at the level of the main nerve (Figure 1 D). Thus, it is possible that neurons project bifurcated fibres towards each neuromast. However, we speculated that since the afferent neurons outnumber neuromasts by a factor of two, more than one neuron could innervate each neuromast. To test this hypothesis, we took advantage of the fact that plasmid DNA injected into eggs is often expressed in a variegated manner. We generated larvæ expressing a membrane-localized red-fluorescent protein in individual afferent neurons, by injecting a cDNA coding for mem-TdTomato under the transcriptional control of the neuronal HuC promoter. Mem-TdTomato decorated the entire neuron, including the somata (Figure 2 A). In the background of the HGn39D transgenic line, this strategy allowed us to resolve individual neurons along their entire length (Figure 2 A–D), and also the thin neurites that form the dendritic arbors beneath the neuromasts (Figure 2 C–G). All the lateralis neurons that expressed mem-TdTomato in HGn39D were also positive for GFP and showed identical dynamics when compared to non-expressors. This indicates that HuC directs expression in afferents and that mem-TdTomato does not affect their behavior. We selected specimens expressing mem-TdTomato in single neurons for detailed analysis by confocal microscopy to find single fibers in 100% of the cases (N = 42) (Figure 2 D), indicating that neurons do not project branched fibers towards any individual neuromast. Together, these results show that each neuromast is contacted by more than one afferent neuron.

### Afferent neurons synapse with hair cells of identical orientation

**Figure 3:**
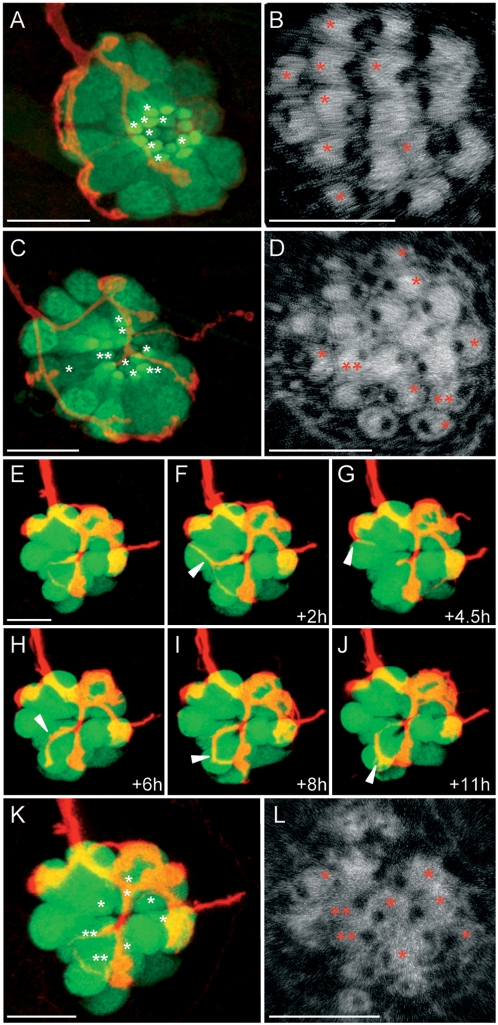
Afferent neurons innervate hair cells of identical polarity. Top views of neuromasts from HuC∶mem-TdTomato injected ET4 fish are depicted. (A–D) maximal projections (A,C) and phalloidin staining (B,D) of neuromasts innervated by an afferent neuron that exclusively innervates hair cells of the same polarity (A–B) and by an afferent neuron that also projects neurites to hair cells of the opposite polarity (C–D). Asterisks indicate the innervated hair cells. Double asterisks point the innervated hair cells of the opposite polarities than the hair cells marked with single asterisks. (E–L) Maximal projection (E–K) and phalloidin staining (L) of the same neuromast. White arrows indicate a non-stable neurite contacting hair cells of opposite polarity (double asterisks) compared to stable synapses (asterisks). Scale bars: 10 µm (A,C, E and K) and 5 µm (B, D and L).

**Figure 4:**
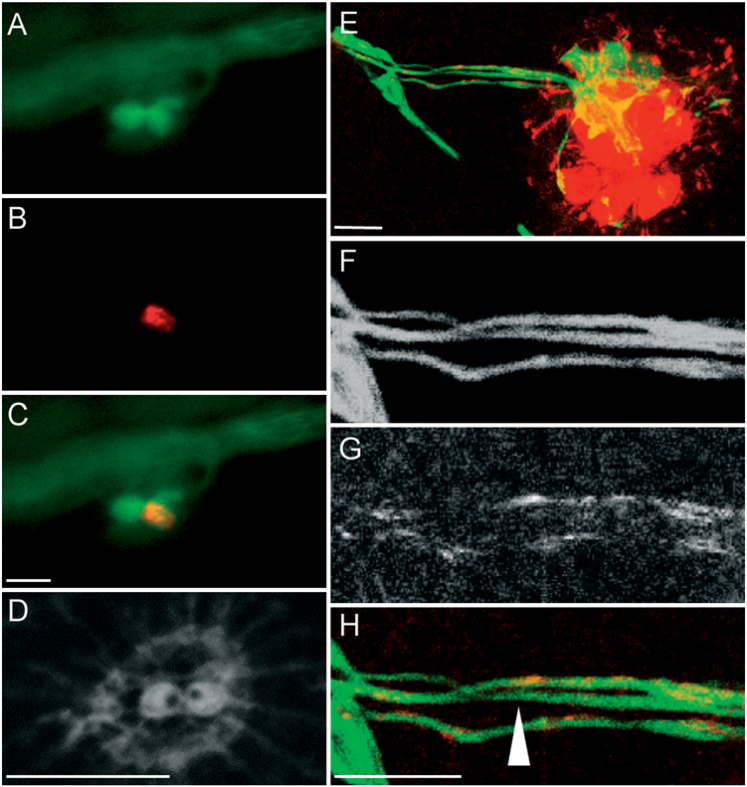
Polarized fluid jet induces incorporation of DiASP in hair cells of selected orientation and is transported to a subset of the afferent neurons. (A–D) DiASP directional incorporation in a neuromast with two hair cells of opposite polarities. (A) GFP expression in HGn39D/SqET4 fish; (B) DiASP incorporation; (C) overlay of GFP expression and DiASP; (D) phalloidin staining reveals the orientation of the hair cells. (E–H) DiASP directional incorporation in a fully developed neuromast doesn't label all the afferent neurons. (E) overlay of HGn39D GFP expression and DiASP incorporation in the hair cells and the afferent neurons ; (F–H) details of the neurons with GFP expression (F), DiASP (G) and overlay of the two (H). The white arrow indicates an afferent neuron that is not labeled by the DiASP. Scale bar: 10 µm.

Our previous observations predict that each neuron innervates only a subset of the constituent hair cells of each lateral-line organ. Because neuromasts harbor two populations of hair cells of opposite polarities, we asked whether there was a relationship between neuronal contacts and the orientation of hair cells. Hair cells in the zebrafish lateral line can be visualized *in vivo* by virtue of specific GFP expression in the SqET4 or brn3c∶GFP transgenic lines [33]–[35]. To determine the contacts of afferent neurons with hair cells of a particular orientation, we performed triple labeling of 7 dpf SqET4 larvæ with fluorescent phalloidin to reveal stereocilia in blue and scatter-labeled afferents with mem-TdTomato (Figure 3 A–L). We performed three-dimensional live imaging analyses in a large number of specimens (N = 25), and found that afferent neurons formed two types of neurites. One type was thin along its entire length, whereas the second type had bulged termini. Thin neurites were long and very motile, extending and retracting very rapidly (Supplementary Movie S1). These neurites often did not contact any hair cell (Figure 2 C). When they established contacts with a hair cell, however, these were short-lived (Figure 3 E–J). In contrast, bulged neurites were shorter, less motile and established stable associations with hair cells (Figure 3 E–K). We found no direct relationship between thin neurites and hair cells of any orientation (Supplementary Movie S1). However, in 12 of 14 cases we observed that bulged neurites associated exclusively with hair cells of identical polarity (Figure 3 A–D and K–L) (Table 1) (Supplementary Movie S2). In the two exceptional cases (4 and 11 of Table 1), a neuron contacted hair cells of both polarities. However, the rare neurites that contacted one or two hair cells of the opposite polarity did not show a prominent bulging at their ends (Supplementary Movie S2). Interestingly, we documented an instance when a bulged neurite with a stable contact with one hair cell (Figure 3 E) projected an extension to contact a second target (arrowhead Figure 3 G). The second contact in this case is with an opposite-polarized hair cell (Figure 3 L). Over time the second contact destabilized for the bulged neurite to retain a stable contact with a single hair cell (Figure 3 K). This analysis highlights the essential need to perform long-time lapse sequence at high temporal resolution to define the dynamics of contacts between hair cells and neurons. Our observations suggest that only bulged neurites form synapses with hair cells. We further corroborated this conclusion by the establishment of an imaging method using DiASP to directly reveal synaptic communication between hair cells of a given polarity and the afferent neurons [11]. When applied in the medium, DiASP enters hair cells by passing directly through mechanically gated transduction channels on the stereocilia and subsequently permeates to the afferent neurons (Figure 1 R–S). DiASP entry into hair cells is blocked when the mechanotransducing channels are closed. To mechanically open transduction channels in hair cells of a selected orientation and close those of the opposite, we applied a fluid stream directed at the neuromasts along the anteroposterior axis of the fish (Supplementary Figure S1). After a brief period of directed water jet to deflected stereocilia, we applied DiASP to the stream. In contrast to the normal loading to all transducing hair cells in the neuromast, fluid jet application of DiASP labeled hair cells whose stereocilia were deflected positively, whereas the cells whose stereocilia were deflected negatively were not labeled (Figure 4 A–D). We reasoned that if each neuron synapses with hair cells of both orientations, DiASP should permeate to all neurons irrespectively of the orientation of the fluid jet. However, when we applied a polarized fluid jet to HGn39D specimens, we observed that DiASP did not label all fibers, suggesting that the unlabelled neurons did not establish synaptic contacts with hair cells of both polarities (Figure 4 E–H). This result is consistent with our previous observations, which together support the conclusion that each afferent neuron forms stable synaptic connections with hair cells of only one orientation.

**Table 1:** Bulged neurites associate exclusively with hair cells of identical polarity (in 12 of the 14 analyzed samples).

Sample	Polarity ‘A’ hair cells innervated	Polarity ‘B’ hair cells innervated
**1**	3	0
**2**	7	0
**3**	6	0
**4**	5	1
**5**	7	0
**6**	6	0
**7**	7	0
**8**	4	0
**9**	4	0
**10**	5	0
**11**	6	2
**12**	8	0
**13**	6	0
**14**	4	0

Each sample corresponds to a 7 dpf SqET4 larvæ neuromast stained with a blue-fluorescent phalloidin to reveal stereocilia and innervated by a single-labeled (mem-TdTomato) afferent neuron. Number of hair cells of different polarities innervated by single afferent neuron is shown in each sample. Polarity ‘A’ is opposite to polarity ‘B’.

### Afferent neurons contact hair cells of identical orientation in adjacent neuromasts

**Figure 5:**
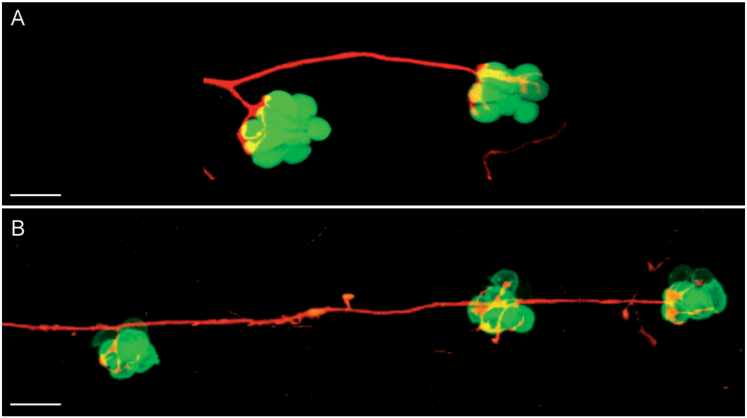
Afferent innervation of adjacent neuromasts. (A–B) Maximal projections of single neurons labelled with mem-TdTomato innervating hair cells of the same polarity of (A) two adjacent neuromasts in an SqET4 transgenic fish and (B) three adjacent neuromasts in a brn3C∶GFP transgenic fish. Those depicted are the terminal neuromasts from a 4 dpf fish. Scale bar: 20 µm.

Mosaic expression of mem-TdTomato showed that some neurons innervate simultaneously two or three adjacent neuromasts (Figure 5). These neurons tended to be the longest: i.e., those that targeted the most caudal organs. We suspected that these neurons would contact hair cells of identical orientation in each neuromast. To test this hypothesis, we examined SqET4 and brn3c∶GFP stable transgenics expressing mem-TdTomato in single neurons. Because hair cells are born in pairs of opposite planar polarity along a single axis creating a line of mirror symmetry, all the cells located anterior to the line of symmetry are polarized posteriorly, whereas the posterior cells adopt an anterior orientation (Figure 4 D) [34]. Thus, the orientation of the hair cells can be predicted from their position with respect to the line of mirror symmetry. When the line of mirror symmetry was evident, we observed that bulged neurites established stable associations exclusively with hair cells on the same side of each neuromast. This indicates that each afferent neuron synapses with hair cells of identical orientation in adjacent neuromasts (Figure 5). Therefore, lateralis afferents can link several organs to form multi-neuromast sensory units.

### Afferent neurons maintain a polarity memory

**Figure 6:**
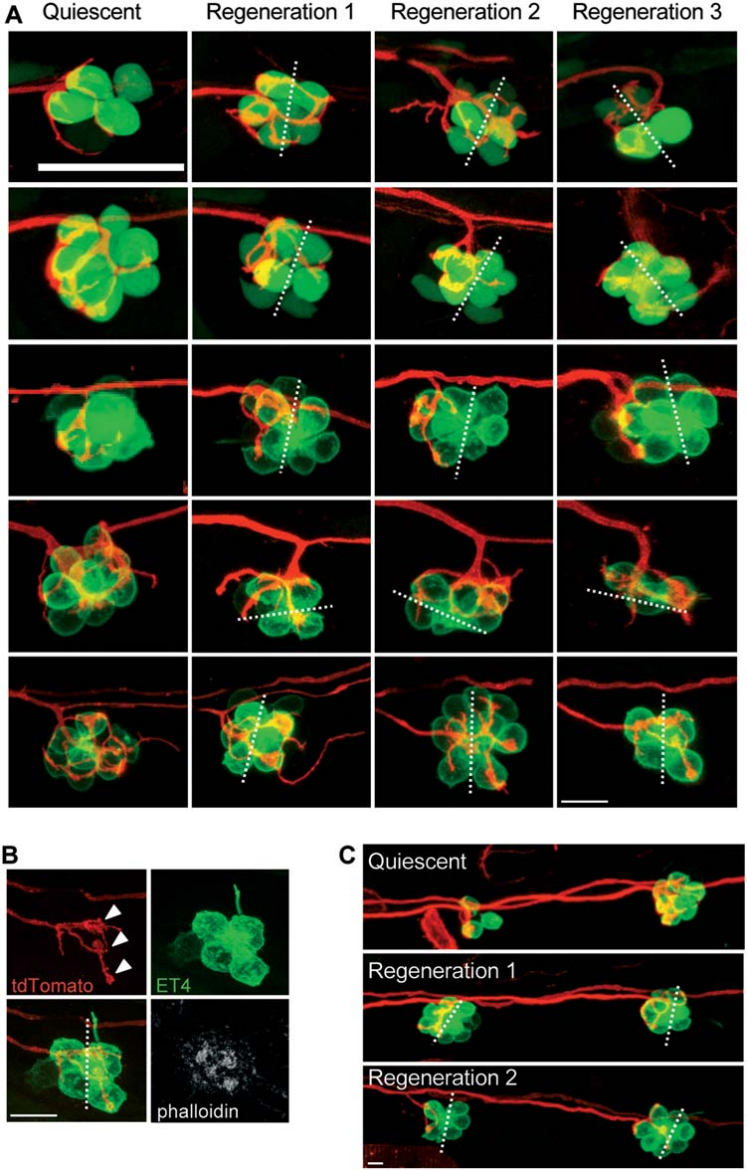
Afferent neurons reinnervate the hair cells of the same polarity after several cycles of ablation and regeneration. (A) Maximal projections of SqET4 (two first lines) or brn3c∶GFP fish neuromasts innervated by a single neuron expressing mem-TdTomato. Confocal z-stacks of the same neuromast have been acquired at 3 dpf (quiescent state, first row) and 20 hours after each of three rounds of neomycin treatments (Regeneration 1, 2 and 3). All rows picture neuromasts with parallel cellular polarity (hair bundles aligned with the anteroposterior axis of the fish body) except the penultimate, which shows a neuromast with perpendicular cellular polarity. (B) Phalloidin staining performed on the last neuromast depicted on A after the third regeneration process. (C) Maximal projections of two brn3c∶GFP adjacent neuromasts innervated by a single neuron expressing mem-TdTomato. Confocal z-stacks of the same neuromasts have been acquired at 3 dpf (quiescent) and 20 hours after each of two rounds of neomycin treatments (Regeneration 1 and 2). Dotted lines represent the axis of polarity of the neuromast. White arrowheads in panel B indicate the bulged neurites establishing contacts with the two hair cells of same polarity. Scale bar: 10 µm.

**Figure 7:**
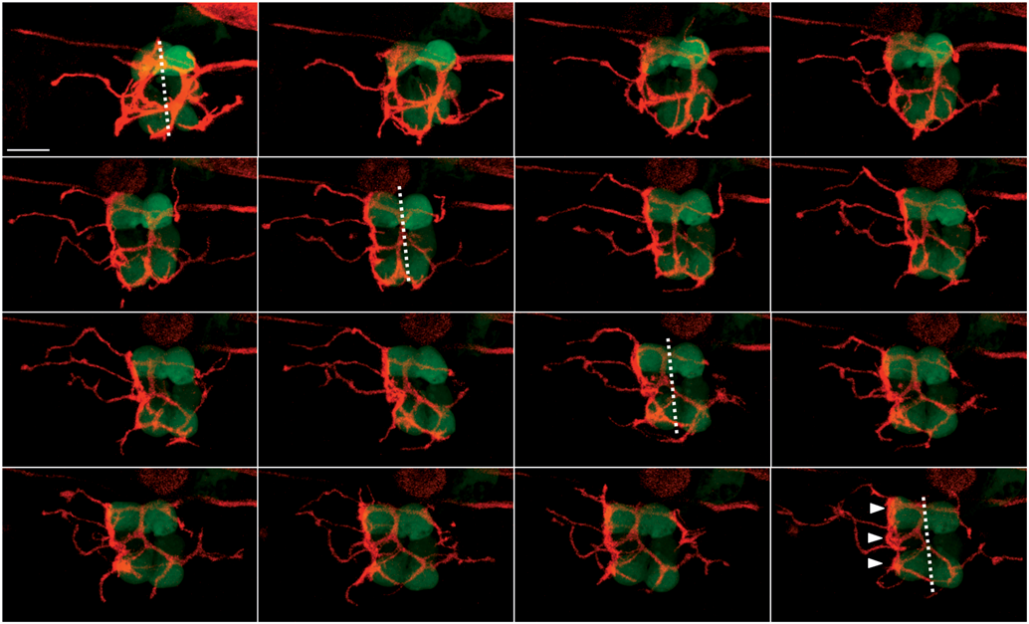
Afferent innervation of regenerating hair cells is a dynamic process. Maximal projections of an SqET4 fish neuromast innervated by an afferent neuron expressing mem-TdTomato. Pictures have been taken every 45 min, starting 20 hours after neomycin treatment. Dotted lines represent the axis of polarity of the neuromast. White arrowheads point to the bulged neurites establishing contacts with the three hair cells of same polarity. Scale bar: 10 µm.

Hair cells of the zebrafish lateral line show robust regeneration after being killed by ototoxic drugs [34], [36]–[37]. Hair cells begin to re-appear around 12 hours after drug treatment to eventually produce a complete anatomical and functional recovery of the organ in 48 hours. This implies that hair cells re-innervate fast during the two-day regeneration period. Neuromasts regenerate hair cells of each polarity in equal numbers, oriented parallel to the neuromast's original axis of planar polarity. The position of a hair cell with respect to the line of mirror symmetry can predict its planar orientation [34]. Therefore, we could unambiguously establish the synaptic choice made by a neuron in living specimens upon several cycles of hair-cell ablation and regeneration. We took advantage of this stereotypic recovery of hair cells to ask whether a neuron would regain contacts with hair cells of identical polarity. We employed the same experimental approach of mosaic expression of mem-TdTomato in the stable transgenic lines SqET4 or brn3c∶GFP. We produced three-dimensional images from neuromasts in living larvæ from confocal Z-stacks before drug treatment, and again imaged the same neuromasts in three dimensions after three consecutive cycles of hair-cell regeneration over a period of 6 days. In every case we observed that after each cycle of regeneration bulged neurites established stable contacts with hair cell lying on the same side of the line of mirror symmetry (Figure 6 A). To establish the orientation of the hair cells directly, we stained sample after the third cycle of regeneration with phalloidin, confirming that hair cells lying on each side of the line of mirror symmetry had identical polarity (Figure 6 B). Neurons selected hair cells of identical orientation irrespectively of the number of neuromasts that they innervated (Figure 6 C). We also produced three-dimensional time-lapse recordings of neuromasts innervated by an afferent neuron starting 20 hours after neomycin treatment. These direct observations at high temporal resolution revealed that hair-cell reinnervation is a highly dynamic process that involves vertiginous neuritogenesis, target recognition and contact stabilization (Figure 7) (Supplementary Movies S3 and S4). We conclude that neurons are strict selectors of planar cell polarity that re-establish synaptic connections with targets of identical orientation during hair-cell regeneration.

## Discussion

In this study we address the stimulus-polarity problem posed by the dual planar polarization of hair cells in the lateral-line system of the zebrafish. We demonstrate that each afferent neuron of the posterior lateral line receives synaptic input exclusively from hair cells that are oriented along the same vector of planar polarity. An important aspect of our conclusions is that they provide an anatomical explanation for the physiological models of signal-polarity resolution by the lateral line.

### Afferent neurons divide the neuromast into synaptic compartments of planar polarity

Hair cells of the superficial lateral line have their mechanosensitive stereocilia embedded into a gelatinous cupula. Thus, the cupula coordinates the deflection of the stereocilia in a manner analogous to that of the tectorial membrane in the mammalian cochlea. Consequently, all hair cells in the neuromast are stimulated simultaneously by water current. The organization of hair cells in opposite polarities suggests that the lateral line can both discriminate a mechanical signals' polarity and its vectorial component. However, a movement of the cupula will depolarize half of the hair cells and hyperpolarize the remaining half simultaneously, creating ambiguity in the resolution of stimulus polarity. How does the system resolve the vector of a mechanical stimulus? One possible mechanism would involve the division of the neuromast into compartments, each including hair cells of identical orientation in analogy to the compartmentalization created by the striola in some inner ears. An apical line of mirror symmetry separating anatomical planar-polarity compartments in neuromasts is evident only transiently during the first few hours of hair-cell development or regeneration [34]. However, hair cells of opposite polarities are intermingled in a mature and fully functional neuromast. Therefore, the neuromast's epithelium appears to lack any anatomical compartmentalization at the apex. The spatial organization of hair-cell apices mirrors that of their bases, suggesting that an anatomical compartmentalization at the basal part of the neuromast is unlikely [34]. An alternative possibility would involve a differential connection between hair cells of each polarity and processing areas in the brain. Such functional compartmentalization would necessitate the innervation of neuromasts by at least two sensory neurons. By scatter-labeling afferent neurons with mem-TdTomato in the HGn39D transgenic line, we were able to dissect the lateral-line neuronal network at the single-cell level. We discovered that each neuromast is contacted by two or more afferent neurons. By performing extensive live imaging recordings of hair cells and neurons in three dimensions, we also showed that neurons arborize extensively and establish synaptic connections with the hair cells. We found no case in which a neuron contacted all the hair cells in a neuromast. By contrast, the majority of the neurons contacted 50% or fewer hair cells, indicating that neuromasts are partitioned into synaptic sub-groups. Therefore, the lateral-line system meets the first essential requirement for functional compartmentalization.

### Each afferent neuron synapses with hair cells of identical orientation

The results above raise the question of whether neurons recognize hair-cell orientation. Because we could define single neurons and their entire structure *in vivo*, we performed live imaging and triple labeling experiments in a large number of specimens. This allowed us to discover that afferent neurons formed two types of neurites. One type, that we call thin neurites, displayed the same diameter along its entire length, was very dynamic and established contacts with hair cells only transiently. The second type of neurites had bulged termini. They were less motile and established stable associations with hair cells, representing synaptic connections. Bulged neurites associated exclusively with hair cells of identical polarity. We considered two possibilities to account for the synaptic association between a neuron and identically-oriented hair cells: that neurons govern the polarization of hair cells, or that neurons recognize hair-cell polarity. In the first case, the afferent system could determine hair-cell orientation. We have previously shown that complete absence of neurons does not affect the polarization of hair cells, ruling out an essential and permissive role of neurons in hair-cell orientation [12]. However, when present neurons could instruct the orientation of hair cells. We consider this unlikely for two reasons. First, hair cells of opposite polarities are born in pairs creating an axis of mirror symmetry in the neuromast, with 100% of the hair cells anterior to this axis placing their kinocilium towards the posterior and *vice versa*. Thus, the orientation of a hair cell is determined by its position relative to its sibling. If planar polarization was determined by a neuron's choice of synaptic partner, the orientation of the stereocilia in sibling hair cells would be stochastic and, consequently, the probability of formation of axes of mirror symmetry would be low. Instead, the axis of mirror symmetry forms in 100% of the cases. Second, it is possible that neuromasts conceal an anatomical compartmentalization on the basal part of the sensory epithelium, which the neurons can use as a landmark to differentiate a hair cell from its sibling. Neurons placed caudally to the axis of mirror symmetry could influence the polarization of the caudal-born hair cell and *vice versa*. However, when pairs of hair cells are produced ectopically at the anterior or posterior poles of the neuromast, siblings still develop opposite polarities. Because we have observed rostral-placed dendrites reaching caudal hair cells, one could argue that neurons could potentially influence the polarization of hair cells at any pole of the neuromast. However, irrespectively of where pairs of hair cells are born, the rostral hair cell always places its kinocilium towards the tail and *vice versa*. These observations argue against a role of afferent neurons in determining the orientation of hair cells. Another possibility is that the efferent neurons orient hair cells. As one efferent neuron can contact all hair cells in a neuromast, not discriminating polarities, a role by the efferent system in the planar polarization of hair cells is unlikely. Taken together, these observations do not support a role of neurons in directing hair-cell orientation.

A second possibility is that afferent neurons detect hair-cell polarity. In a recent study, Nagiel and colleagues analyzed this issue [38]. The authors used identical strategies to express a membrane targeted red-fluorescent protein to afferent neurons, and also the same transgenic strains expressing the GFP in hair cells of the lateral line. In addition, comparable stages of development, and identical methods to ablate hair cells in neuromasts were used. These similarities allow a straightforward comparison of the two studies, which for the most part report identical results. However, Nagiel and colleagues used sophisticated statistical analyses of static light-microscopy images, to conclude that although afferent neurons can recognize hair-cell polarity, erroneous synapses occur frequently. These authors suggested that afferents act as “biased selectors” of hair-cell orientation. We have employed direct, *in vivo* long-term three-dimensional imaging at high temporal resolution, to find that the system is much more complex. Neurons frequently project neurites to contact hair cells. However, most of the contacts are transient, and do not necessarily represent synapses. Neurites displaying such highly dynamic exploratory behavior could use a trial-and-error mechanism to probe hair-cell orientation. This implies that at any given moment, only a small proportion of the contacts between these two cell types are functional synapses. Therefore, the erroneous synapses reported by Nagiel and colleagues likely represent non-synaptic transient interactions [38]. In addition, using polarized DiASP jet application to directly reveal synaptic connections, we showed that neurons do not establish erroneous synapses. One potential caveat of our experiment is that wrong-synapsed hair cells can be few, not allowing enough DiASP to permeate into neurons above the detection limit of the microscope. However, these results taken together with our live imaging analyses and the electrophysiological studies in zebrafish and other species do not support the biased selection model [21]–[22], [38]–[39]. One important aspect of our conclusion that afferent neurons are strict selector of polarity is that it can reconcile the anatomical and electrophysiological models of signal-polarity resolution by the lateral line [21]–[22], [39].

How is the strict selection achieved? One possibility is that the efferent system instructs afferent neurons to select the correct target. Efferent neurons in the lateral line do not contact afferents directly. Thus, a communication between the two neuronal types would require the intervention of hair cells. Because one efferent neuron can contact all the hair cells in a neuromast, not discriminating polarities, its input would lack asymmetry. We conclude that a role of the efferent system in hair-cell planar-polarity recognition by afferents is unlikely. There is no evidence of a molecule that is asymmetrically segregated between sibling hair cells, which appear identical albeit opposite polarized. Therefore, another possibility is that the selection of hair-cell polarities by afferent neurons is a two-step process. We favor this second model because neuromasts initially develop a single pair of hair cells of opposite polarities. Afferents would have choice between only two targets. Based on this data, we suggest that the initial contact between neurons and hair cells is stochastic. Contacts would subsequently be refined by an evoked-activity-dependent mechanism to stabilize a one-to-one synaptic connection between each neuron and only one of the first two hair cells. Neurons would continue to explore for targets as new hair cells develop using a trial-and-error mechanism. As hair cells continue to appear progressively and in pairs of opposite polarity, afferents would again choose a new target between only two possibilities. The same activity-dependent mechanism would now play a different role: to instruct each neuron to form stable synapses only with hair cells that are polarized along the same vector of the neuron's first target. This process iterates while the neuromast augments its hair-cell population. Because neurons constantly extend neurites to explore for new targets, the process must be maintained throughout life to prevent the establishment of synapses with opposite-oriented hair cells. The mechanism of “trial-and-error exploration” and subsequent “activity-dependent refinement” may be analogous to that found in the mammalian cochlea, where the contacts of afferent neurons gradually retract from several to just one hair cell during the second postnatal week [40]–[41]. One hypothesis that derives from these conclusions is that animals with compromised mechanoreception or mechanotransduction would permit individual afferent neurons to stabilize synapses with hair cells of both polarities. The existence of several profoundly deaf zebrafish strains will allow this hypothesis to be tested in the future.

### Neurons can form synapses with hair cells of identical orientation in adjacent neuromasts

When labeling individual neurons with mem-TdTomato, we observed neurons that synapsed with hair cells in adjacent neuromasts. This could group several neuromasts into a single sensory unit. However, for these multi-neuromast units to work efficiently a condition *sine-qua-non* is that each neuron forms synapses with hair cells of identical orientation in all neuromast. Indeed, single-neuron imaging in SqET4 transgenics showed that each afferent that innervated at least two neuromasts contacted hair cells of identical orientation. These results suggest that while the cupula coordinates the mechanical stimulation of all the hair cell in each neuromast, the neurons decouple hair cells based on orientation in each neuromast and, simultaneously, couple hair cells of identical orientation in adjacent neuromasts. Our results demonstrate striking similarities with the innervation of identical populations of hair cells in all the neuromasts forming a stitch in *Xenopus*, implying that the mechanisms governing the formation of multi-neuromast sensory units is conserved across species [21]–[22]. We wondered about the selective pressure for the evolution of multi-neuromast units. It has been proposed that the resting activity (summation of spontaneous and evoked activities) of an afferent neuron increases with the number of the neuromasts it contacts [42]. But because the spontaneous activity is independent of the number of neuromasts, only the evoked activity would increase by the recruitment of more neuromasts by the afferent neurons [43]. Therefore, multi-neuromast sensory units should permit both the amplification of the mechanical signal and a reduction of noise in the system without the need to increase the number of hair cells per neuromast, which would otherwise reduce sensitivity due to the larger resistance to cupula bending by more numerous hair bundles [44]. This thesis takes support in the observation that while most organs increase their size as the fish grows, neuromasts increase in number but remain small.

### Afferent neurons contact identically oriented targets during hair-cell regeneration

Signals that stabilize or destabilize synapses to create functional connectivity are especially relevant when emanating from sensory epithelia undergoing remodeling during changes in demand, or while repairing after damage. We have previously demonstrated that hair cells of the zebrafish lateral line, when killed by ototoxic drugs, begin to regenerate around 12 hours after treatment to eventually produce a complete anatomical and functional recovery of the organ in 48 hours [34]. This implies that hair cells re-innervate fast during the two-day regeneration period. By performing iterative cycles of hair-cell ablation and regeneration in doubly transgenic larva, we discovered that the initial choice of a synaptic partner made by neurons during embryonic development is retained during hair-cell regeneration. We currently cannot offer a satisfactory explanation for this observation. However, we also observed a preferential segregation of dendritic arbors to either pole of a quiescent neuromast (Figure 2 E–G), and that dendries do not fully retract after hair-cell death. This intrinsic asymmetry of the dendritic arbor could be used by afferent neurons to perform an initially stochastic but biased selection of the first target. Hair-cell activity would subsequently stabilize the synaptic connections over a period of several hours. Our *in vivo* imaging analyses reveal that the matching of a neuron with hair cells of identical orientation is evident only when the innervation process is observed directly at high temporal-resolution over long periods.

In conclusion, our studies indicate that lateralis afferent neurons ultimately are strict selectors of hair-cell polarity. The model that emerges from our results suggests that in the absence of anatomical compartments in the sensory epithelium, the neurons partition the neuromast into synaptic compartments of planar polarity, effectively allowing this sensory system to discriminate the vectorial component of a mechanical stimulus.

## Materials and Methods

### Zebrafish strains and husbandry

Zebrafish were maintained under standardized conditions and experiments were conducted in accordance with protocols approved by the PRBB's Ethical Committee of Animal Experimentation. Naturally spawned eggs were collected, cleaned, and maintained in system water at 28.5°C at a density of 50 per 85 mm Petri dish. Embryos were staged according to Kimmel *et al.* (1995) [45]. SqET4 and brn3c∶GFP transgenic animals expressing GFP were obtained, respectively, from V. Korzh and H. Baier. HGn39D was generated by random integration of an enhancer-trap transgene [28]. Hair-cell ablation was performed by treating zebrafish 4–7 dpf larvæ with 250 mM neomycin sulfate (Sigma) for 45 minutes at room temperature, and then let to recover in system water at 28.5°C.

### Plasmid DNA constructs and injections

The HuC∶farnesylated-TdTomato (HuC∶mem-TdTomato) construct was obtained using the “Tol2 kit”. Entry vectors were generated as described in the Invitrogen Multisite Gateway manual. PCR were performed using primers to add *att* sites onto the end of DNA fragments, using Platinum *Pfx* (Invitrogen). For the generation of the 5′ entry clone containing the HuC promoter, (using pDONR P4-P1R), the forward PCR primer containing an *attB4* site and the reverse primer containing a reverse *attB1* site were used:

Forward: 5′-GGGGACAACTTTGTATAGAAAAGTTGTAATACGACTCACTAGTGG-3′
Reverse: 5′-GGGGACTGCTTTTTTGTACAAACTTGCCTCTTGACGTACAAAGATGA-3′

For the generation of the middle entry clone containing the TdTomato cDNA (using pDONR 221), the forward PCR primer containing an *attB1* site and the reverse primer containing a reverse *attB2* site were used:

Forward: 5′-GGGGACAAGTTTGTACAAAAAAGCAGGCTCCACCATGGTGAGCAAGGGCGAGGA-3′
Reverse: 5′-GGGGACCACTTTGTACAAGAAAGCTGGGTGCTTGTACAGCTCGTCCATGC-3′

For generation of the 3′-entry clone containing the farnelysation domain of the Ras protein (using pDONR P2R-P3), the forward PCR primer containing an *attB2* site and the reverse primer containing a reverse *attB3* site were used:

Forward: 5′-GGGGACAGCTTTCTTGTACAAAGTGGCCGGAGGAGGAAGATCTAAGCT-3′
Reverse 5′-GGGGACAACTTTGTATAATAAAGTTGCTCAGGAGAGCACACACTTGC-3′

PCR products were purified using the Qiaquick gel extraction kit (Qiagen). BP and LR reaction were performed as described [46].

5′ capped sense RNAs were synthesized using a construct encoding the transposase and the mMessage mMachine kit (Ambion). 20 pg of the HuC∶TdTomato-farn DNA construct and 20 pg of the transposase sRNA were simultaneously injected into embryos at the one or two-cell stage.

### Labeling procedures

For vital labeling of hair cells, zebrafish larvæ were immersed in a 500 µM solution of DiASP for three minutes at room temperature in the dark. Treated larvæ were washed briefly to remove excess fluorophore, anæsthetized in 3-aminobenzoic acid ethyl ester solution (Sigma), mounted on a glass slide, and aligned using a hair loop. For immunohistochemistry, larvæ were fixed overnight at 4°C in a solution of 4% paraformaldehyde in phosphate-buffered saline (PBS) solution containing 1% Tween-20. After fixation, samples were washed in the same solution without fixative and blocked at room temperature with 10% bovine serum albumin. Primary- and secondary-antibody incubations were conducted overnight at 4°C in PBS with 0.2% Tween-20. Labeling with fluorescent phalloidin was done as published using Alexa-Fluor 350 or Alexa-Fluor-647 phalloidin 1∶25 (Invitrogen) [12]. Primary antisera and monoclonal antibodies were used at the following dilutions: rabbit anti-Tyrosine hydroxylase, 1/1000 (Pel-Freeze); mouse monoclonal antibody HCS-1, 1/20; and rabbit anti-Cav1.3a (Gem25.2), 1/500. Texas Red-labeled donkey anti-mouse, and -rabbit immunoglobin secondary antibodies (Jackson Labs) were used at a dilution of 1/150. Fluid jet application of DiASP was done on anæsthetized zebrafish larvæ placed into a 1 mm width well in an agarose disk. The tail of the fish was attached to the agarose by a 5 mm long hair. A 10–15 seconds stream of E3 medium was applied to the fish from its tail along its anteroposterior axis using a wash bottle (Nalgene) with a 1 mm diameter aperture. During the continuous E3 medium stream, DiASP was applied during 1–3 seconds near the tail of the fish to the directional fluid stream. DiASP was applied at a 50 mM concentration using a pipette tip (Eppendorf).

### Live imaging

We created genetic mosaics to scatter-label afferent neurons by injecting a plasmid coding for a membrane-targeted TdTomato construct under the transcriptional control of the neuronal HuC promoter (HuC∶mem-TdTomato) into fertilized eggs of stable transgenic HGn39D, SqET4 or brn3c∶GFP animals. We screened embryos that expressed the GFP with a stereomicroscope under ultraviolet light, and selected those expressing mem-TdTomato in single neurons. Embryos were let to grow in system water at 28.5°C in 85 mm Petri dish for four or five days in the dark. Neuromasts contacted by a single red neuron were selected for long term live imaging by laser-scanning confocal microscopy. For this purpose, zebrafish larvæ were mounted on the coverslip of a glass-bottom dish (Mattek), covered with 1% low-melting-point agarose in a 610 µM solution of the anæsthetic 3-aminobenzoic acid ethyl ester, and imaged immediately. Images were acquired with a Leica TCS SPE confocal microscope with a 40× oil immersion objective. Z-stacks were acquired at 0.8 mm intervals, imaging GFP (488 nm excitation, 500–550 nm emission) and TdTomato (532 nm excitation, 570–630 nm emission). We generated three-dimensional images over time (4-D images) for periods ranging from 3 to 24 hours and different temporal resolutions.

### Scoring of hair-cell orientation

We conducted analyses of hair-cell orientation on neuromasts of the posterior lateral line. Actin-labeled neuromasts were imaged using a confocal microscope. Orientations of hair cells in neuromasts were determined by drawing a line connecting the kinocilum to the opposite edge of the hair bundle.

## Supporting Information

Figure S1 DiASP application during directional E3 medium stream. (A) Scheme depicting the placement of: the fish, the hair (brown line) used to attach it into the well, the wash bottle with E3 medium (in blue) and the pippette tip with DiASP (in red). (B) Cartoon depicting the incorporation of DiASP (orange) in only hair cells of one polarity (2) and not the others (1). Black Arrows indicate the direction of the fluid stream along the anteroposterior axis of the fish.
(8.99 MB TIF)

Movie S1 This time-lapse sequence shows the dynamics of innervation of a neuromast at a quiescent state (neuromast of figure 3 E–L). A single mem-TdTomato labeled neuron is followed in a sqET4 transgenic fish. We can observe thin neurites that are long and very motile, extending and retracting very rapidly. Their contacts with the hair cells are short-lived. In contrast, bulged neurites are shorter, less motile and establish stable associations with hair cells. The larva was mounted in 1% low-melting-point agarose, maintained at 21°C and images were acquired with a Leica TCS SPE confocal microscope with a 40× oil immersion objective every 30 minutes during 10,5 hours. Z-stacks were acquired at 0.8 µm intervals.
(0.19 MB MOV)

Movie S2 Animation showing the 3D reconstruction of an ET4 neuromast innervated by a mem-tdTomato labelled afferent neuron exclusively associated to hair cells of identical polarity. This neuromast is the sample 12 in Table 1. Asterisks indicate the bulged neurites that contact the basal domain of the hair cells. A phalloidin staining of this neuromast is shown in Figure 3B.
(0.88 MB MOV)

Movie S3 This time-lapse sequence shows the dynamics of re-innervation of a neuromast during regeneration (Figure 7). A single mem-tdTomato-labeled neuron is followed in a SqET4 transgenic fish. This sequence clearly shows the initial trial-and-error exploratory behavior of the neuron is refined over time. The dendritic arbor re-localizes to establish synaptic contacts exclusively with all the hair cells on the same side of the line of mirror symmetry. The movie starts 20 hours after a 45 min exposure of the animal to 250 µM of neomycin. The larva was mounted in 1% low-melting-point agarose, maintained at 21°C and images were acquired with a Leica TCS SPE confocal microscope with a 40× oil immersion objective every 5 minutes during 17 hours. Z-stacks were acquired at 0.8 µm intervals.
(1.57 MB MOV)

Movie S4 Animation showing the 3-D reconstruction of the SqET4 neuromast innervated by a mem-TdTomato-labeled afferent neuron shown in figure 7 and at the last frame of the Supplementary Movie 3.
(1.12 MB MOV)
