# Supplementary figures and images for: Afferent Neurons of the Zebrafish Lateral Line Are Strict Selectors of Hair-Cell Orientation
Source: PLoS One. 2009 Feb 18;4(2):e4477. doi: 10.1371/journal.pone.0004477 (PMC2637426; doi:10.1371/journal.pone.0004477)

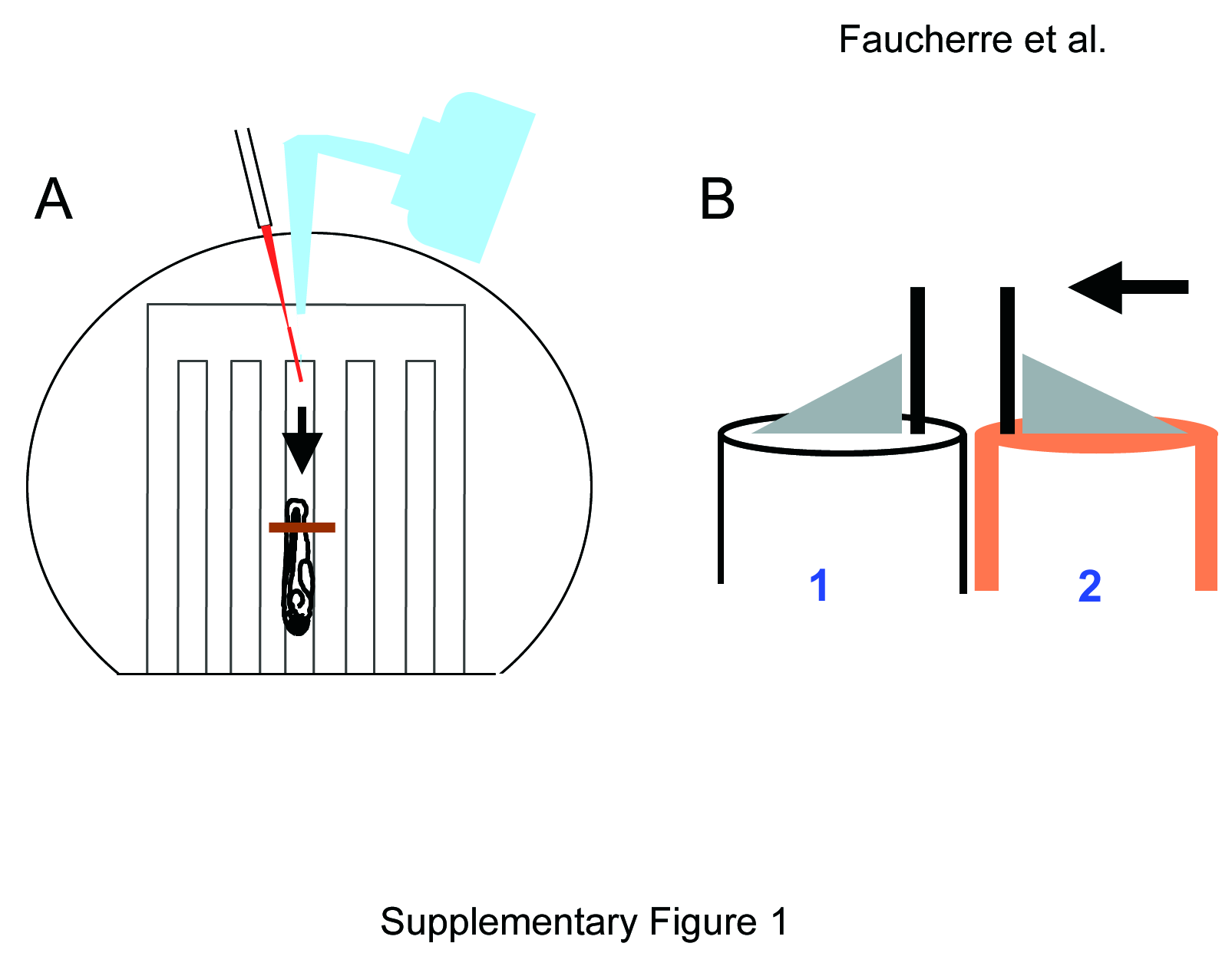

Supplement: Figure S1 — DiASP application during directional E3 medium stream. (A) Scheme depicting the placement of: the fish, the hair (brown line) used to attach it into the well, the wash bottle with E3 medium (in blue) and the pippette tip with DiASP (in red). (B) Cartoon depicting the incorporation of DiASP (orange) in only hair cells of one polarity (2) and not the others (1). Black Arrows indicate the direction of the fluid stream along the anteroposterior axis of the fish. (8.99 MB TIF) [file pone.0004477.s001.tif]
